# Structures of Class I and Class II Transcription Complexes Reveal the Molecular Basis of RamA‐Dependent Transcription Activation

**DOI:** 10.1002/advs.202103669

**Published:** 2021-11-10

**Authors:** Min Hao, Fuzhou Ye, Milija Jovanovic, Ioly Kotta‐Loizou, Qingqing Xu, Xiaohua Qin, Martin Buck, Xiaodong Zhang, Minggui Wang

**Affiliations:** ^1^ Institute of Antibiotics Huashan Hospital Fudan University Shanghai 200040 China; ^2^ Key Laboratory of Clinical Pharmacology of Antibiotics National Health Commission of the People's Republic of China Shanghai 200040 China; ^3^ Section of Structural Biology Department of Infectious Diseases Imperial College London London SW7 2AZ UK; ^4^ Department of Life Sciences Imperial College London London SW7 2AZ UK

**Keywords:** antibiotic resistance, class I and class II activators, cryoEM structures, RamA, RNAP‐*σ* ^70^

## Abstract

Transcription activator RamA is linked to multidrug resistance of *Klebsiella pneumoniae* through controlling genes that encode efflux pumps (*acrA*) and porin‐regulating antisense RNA (*micF*). In bacteria, *σ* ^70^, together with activators, controls the majority of genes by recruiting RNA polymerase (RNAP) to the promoter regions. RNAP and *σ* ^70^ form a holoenzyme that recognizes ‐35 and ‐10 promoter DNA consensus sites. Many activators bind upstream from the holoenzyme and can be broadly divided into two classes. RamA acts as a class I activator on *acrA* and class II activator on *micF*, respectively. The authors present biochemical and structural data on RamA in complex with RNAP‐*σ* ^70^ at the two promoters and the data reveal the molecular basis for how RamA assembles and interacts with core RNAP and activates transcription that contributes to antibiotic resistance. Further, comparing with CAP/TAP complexes reveals common and activator‐specific features in activator binding and uncovers distinct roles of the two C‐terminal domains of RNAP *α* subunit.

## Graphical abstract

Transcription activator RamA is linked to multidrug resistance of *Klebsiella* *pneumoniae*. This study reveals the molecular basis for how RamA interacts with RNA polymerase (RNAP) and activates transcription that contributes to antibiotic resistance. Further, comparing with catabolite activator protein/thermophilic CAP homolog ( CAP/TAP) complexes reveals common and activator‐specific features in activator bindings and uncovers distinct roles of the two C‐terminal domains of RNAP.

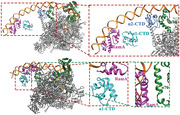

## 1 Introduction

Multidrug resistance in bacteria is a global health threat. *Klebsiella pneumoniae* is one of the major bacterial species responsible for nosocomial infections. The emergence of multidrug‐resistant *K. pneumoniae* has become one of the most problematic issues in the current management of bacterial infections. RamA is an intrinsic regulator in *K. pneumoniae*, belonging to the AraC family of transcription factors and conferring a multidrug resistance phenotype.^[^ ^1^ ^]^ Several studies indicated the important relationship between *ramA* overexpression and antibiotics resistance, particularly tigecycline resistance, in clinical isolates.^[^ ^2^ ^]^ Our previous study identified RamA as a transcription activator of *acrAB* and *oqxAB* genes encoding two efflux pumps, which contribute to antibiotic resistance of *K. pneumoniae*.^[^ ^3^, ^4^ ^]^ RamA is also demonstrated to activate the transcription of antisense RNA *micF*.^[^ ^5^ ^]^ Furthermore, our another study found *micF* can bind to *ompF* mRNA resulting in decreased production of the OmpF porin in *Klebsiella aerogenes* resulted in carbapenem resistance.^[^ ^6^ ^]^ Compared with other AraC family transcription regulators in *K. pneumoniae* such as MarA and SoxS, RamA played a more prominent role in antibiotic resistance in *K. pneumoniae*.^[^ ^7^ ^]^ AraC family proteins are highly conserved small proteins that contain two helix‐turn‐helix (HTH) motifs^[^ ^8^ ^]^ and bind to a degenerate consensus sequence called marbox with considerable overlap in the genes they control.^[^ ^9^ ^]^ The structure of RamA still remains unknown, but the structure of homologous protein MarA has been solved. The crystal structure of MarA‐DNA complex reveals that MarA binds DNA as a monomer, to adjacent segments of the promoter DNA major grooves through the two HTH motifs of MarA.^[^ ^10^ ^]^

Bacterial RNA polymerase (RNAP) is a multisubunit complex consisting of two *α*‐subunits (*α*1 and *α*2), *β*, *β*’ and *ω*.  *α*−units have two domains connected by highly flexible linkers, the N‐terminal domains (NTD) of the two *α*‐subunits interact with each other and form part of the RNAP core while the C‐terminal domains (CTD) are flexible in the absence of activators and are involved in activator binding.^[^ ^11^, ^12^ ^]^ *α*1 and *α*2  are identical in protein sequence with *α*1 being closely associated with *β* while *α*2 closely associated with *β*’ in the RNAP core structure.^[^ ^13^ ^]^  *σ* factors recruit RNAP to the specific promoter, thus conferring promoter specificity, with *σ* ^70^ controlling the majority of genes.^[^ ^14^ ^]^ AraC family members are simple *σ* ^70^‐dependent activators, which are divided into class I and class II. Class I activators typically bind at DNA sequences upstream from the core promoter element whereas class II activators bind at sites overlapping with the core promoter elements.^[^ ^12^, ^15^ ^]^ The majority of *σ* ^70^ activators function through enhanced recruitment and retention of RNAP to the promoter DNA.

Structures of the catabolite activator protein (CAP) in complex with RNAP‐*σ* ^70^ open complex at the *lac* promoter (class I)^[^ ^16^, ^17^ ^]^ and at the *gal* promoter (class II)^[^ ^18^ ^]^ as well as a thermophilic CAP homolog (TAP) in complex with RNAP‐*σ* ^A^ open complex at a class II promoter^[^ ^19^ ^]^ have revealed the structural arrangement of CAP/TAP on the two classes of promoters and confirmed many decades of biochemical and genetic studies, providing a structural basis on how they activate transcription at these promoters. Despite these advances, there is a lack of structural information on how other activators arrange and activate transcription since specific domains of CAP/TAP are involved in interacting with RNAP‐*σ* ^70^/*σ* ^A^, *α*‐CTD, and DNA. Are there common features that are shared among different activator proteins? What are the specific features that are unique to individual activators, which vary in size and oligomeric state?

We thus set out to characterize RamA in the context of *acrAB* and *micF* promoters, providing biochemical and structural data on how RamA regulates transcription of class I and class II promoters that contribute to antibiotic resistance. In addition, RamA is a small protein (≈110 amino acids) and acts as a monomer, while CAP is larger, almost twice that of RamA (≈210 amino acids) and acts as a dimer. By comparing these different activator class I/class II complexes, why RamA acts as a weaker activator compared with CAP was revealed. Furthermore, our studies reveal common features in *σ* ^70^ and *α*‐CTD that are utilized in activator binding as well as versatile strategies utilized by activator proteins in recruiting/enhancing RNAP‐*σ* ^70^ to promoter sites.

## 2 Results

### 2.1 RamA Enhances Transcription Activities In Vitro and In Vivo

**Figure 1:**
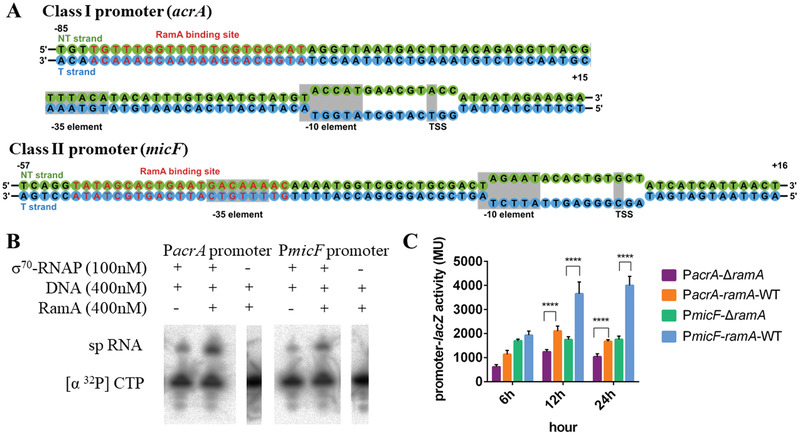
RamA increases transcription at both Class I (*acrA*) and Class II (*micF*) promoters. A) Sequences of Class I and Class II promoters used in this study. B) spRNA assays in the presence of RNAP‐*σ* ^70^ and promoter DNA with and without RamA. C) In vivo transcription as measured by LacZ activities under either Class I or Class II promoter. **** means *P* < 0.0001.

Since AraC family of transcription factors bind to the marbox sequences (TTTXXXXXXXCGTG), we analyzed promoter sequences upstream of the *acrA* and *micF* transcription start sites and identified a RamA binding site at ≈‐71.5 for the *acrA* promoter and at ≈‐41 for the *micF* promoter, which corresponds to class I and class II activator binding sites respectively (**Figure** **1**A). In order to establish that RamA directly acts on *acrAB* and *micF* genes, in vitro short primer (sp) RNA assays and in vivo transcription assay were carried out using *acrA* and *micF* promoters. In vitro assay showed that RamA increases the level of transcription by approximately twofold for both promoters (Figure 1B). In vivo transcription assay showed that the transcription activities are stimulated approximately twofolds by RamA on both *acrA* and *micF* promoters compared with the absence of RamA at 12 or 24 h (Figure 1C).

### 2.2 Structures of RamA‐RNAP‐*σ* ^70^‐*acrA*/*micF* Promoter Complex

**Figure 2:**
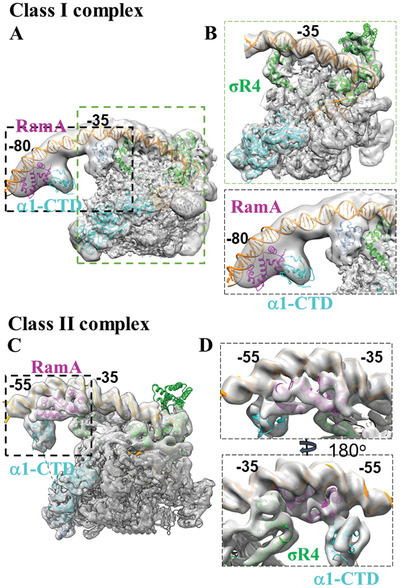
CryoEM reconstructions of RamA in complex with RNAP‐*σ* ^70^ at A–B) Class I and C–D) Class II promoters. Structural models of RNAP‐*σ* ^70^ open complex (from 6CA0) can be fitted into both reconstructions. B) Enlarged regions as boxed in (A) showing *σ* ^70^ region 4 (*σ*R4) at ‐35 promoter DNA and extra density upstream around ‐80 promoter region. D) Front and back views around RamA (boxed region in C) with structural models fitted in, showing RamA binding and interactions with DNA, *σ*R4 and *α*‐CTD. RNAP—gray, *σ* ^70^—green, *α*1‐CTD—cyan, *α*2‐CTD—light blue, RamA—magenta, promoter DNA—orange.

In order to provide the molecular and structural basis for RamA's roles in activating transcription, cryo electron microscopy (cryoEM) single‐particle reconstruction was used to study the structures of RamA‐RNAP‐*σ* ^70^ ‐*acrAB* (class I activator)/*micF* (class II activator) promoter complex (**Figure** **2**, Figures S1–S4, Supporting Information). Since *σ* ^70^‐dependent promoter complexes are unstable as closed complexes, mismatches were introduced within the transcription bubble (Figure 1A) to ensure a stable open promoter complex is formed. The overall resolution of the two reconstructions is similar. However, the electron density around the RamA is better resolved in the class II complex compared to class I (comparing Figure 2A,C). The molecular model of RamA/*α*‐CTD complex obtained from class II reconstruction has been used to fit into the class I complex reconstruction.

The structure of RamA‐Class I promoter complex at the *acrA* promoter was solved at a global resolution at 4.1 Å (Figures S1 and S3, Supporting Information). The best‐resolved region is in RNAP‐*σ* ^70^ (Figure S5, Supporting Information). Indeed, the *E. coli* RNAP‐*σ* ^70^ open complex model (PDB code 6CA0) could fit into the main body of the electron density covering DNA from ‐45 to +15 (Figure 2A,B). There is density for upstream DNA to ‐85 and there is additional density that could account for RamA/*α*‐CTD (Figure 2A,B lower panel, Figure S6, Supporting Information). Due to the flexibility between the promoter DNA where RamA binds and the rest of RNAP‐*σ* ^70^ holoenzyme, the reconstruction is of insufficient quality to allow unique fitting of structural models of RamA and/or *α*‐CTD into the density. Nevertheless, there is connecting density between *α*‐NTD and the density upstream, suggesting that this region could contain *α*‐CTD (Figure S7A, Supporting Information, Figure 2A). Given that *α*‐CTD is likely to coexist with RamA at the promoter region, the RamA/*α*‐CTD structural model obtained from class II reconstruction (see below) was placed into the extra density around ‐80 promoter region with *α*‐CTD downstream of RamA (Figure 2A,B). There is also additional density immediately upstream of ‐35 unaccounted for (Figure 2B lower panel). Lowering the display threshold of the electron density reveals that this region is connected to *α*‐NTD, thus this density is likely to be *α*‐CTD. Based on the density that connects to RNAP core (Figure S7A, Supporting Information), we have assigned that *α*1‐CTD interacts with RamA whereas *α*2‐CTD resides next to *σ* ^70^ (Figure 2). *α*2‐CTD is mobile in the absence of activators as observed in published open promoter complex structures. The spatial arrangement of RamA in relationship to the two *α*‐CTD domains is consistent with the established model of class I activator, where activator binds upstream of *α*‐CTD.

The structure of RamA‐class II activator complex at the *micF* promoter has an overall resolution of 4.0 Å (Figure 2C, Figures S2 and S4, Supporting Information). The best‐resolved region in the RNAP‐*σ* ^70^ shows clear density for side chains (Figure S5, Supporting Information). The RNAP‐*σ* ^70^‐open promoter complex structure (PDB code 6CA0) readily fitted into the main body of the density map. There is extra density further upstream of ‐35, which shows clear density for DNA, including major and minor grooves and density that could account for RamA and *α*‐CTD (Figure 2C,D, boxed regions, Figure S6, Supporting Information). In order to obtain molecular details of RamA interactions, MarA‐DNA crystal structure was used to generate a homology model of RamA‐DNA using Phyre2 due to their high sequence conservation (46% sequence identity, ^[^ ^10^ ^]^ Figure S8, Supporting Information). Indeed, the RamA‐DNA structural model could be fitted convincingly into the density, including accurate positioning of secondary structures and those of DNA (Figure 2D). In this structural model, RamA binds at promoter centered around ‐35 and ‐45 with one HTH domain binding to the groove of the ‐35 element, just upstream from the *σ* ^70^ R4 (*σ*R4) (Figure 2D). RamA thus interacts with *σ*R4 (Figure 2D). There is still extra density that could accommodate an *α*‐CTD (Figure 2D, Figure S6, Supporting Information), upstream of and contacting RamA as well as DNA at ‐55 position. By lowering the display threshold, the electron density map suggests that this *α*‐CTD most likely is connected to *α*1‐NTD of RNAP (thus is *α*1‐CTD) although resolution is insufficient to confirm this (Figure S7B, Supporting Information).

### 2.3 Effects of RamA Mutants on Promoter Activities In Vivo

**Figure 3:**
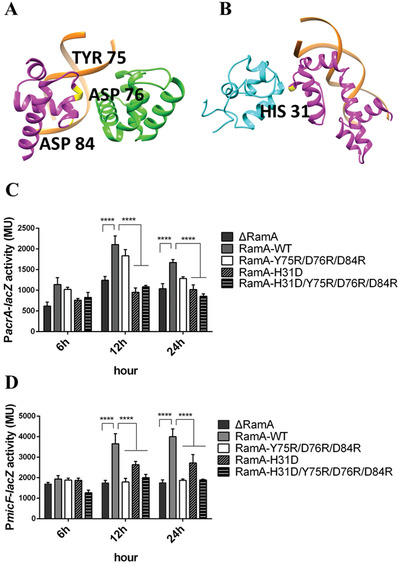
Mutagenesis to investigate the effects of the interacting interfaces. A) Interfaces between RamA and *σ* ^70^ and residues mutated (Y75D/D76R/D84R) to disrupt it and B) Interface between RamA and *α*‐CTD in class II activator complex and residues mutated (H31D) to perturb it. C,D) RamA mutations reduce the ability of RamA to activate transcription at Class I (C) and Class II (D) promoters. **** means *P* < 0.0001.

The structural model of class II complex suggests that H31, Y75, D176, and D84 could be involved in interactions with *α*‐CTD and *σ* ^70^ R4 respectively (**Figure** **3**A,B). To investigate these interfaces, we mutated these residues by reversing their charges. The ability in activating the transcription of wildtype (WT) and mutant RamA was then measured by in vivo transcription assay. For *acrA* class‐I promoter, the transcription activities of RamA H31D and RamA H31D/Y75R/D76R/D84R declined significantly compared with RamA WT (*P* < 0.0001) and had similar activities with the absence of RamA group. The transcription activity of RamA Y75R/D76R/D84R only decreased slightly compared with RamA WT (Figure 3C). For *micF* class‐II promoter, the transcription activities of RamA H31D, RamA Y75R/D76R/D84R and RamA H31D/Y75R/D76R/D84R all decreased significantly compared with RamA WT (*P* < 0.0001). But only RamA Y75R/D76R/D84R and RamA H31D/Y75R/D76R/D84R reached the same low transcription level seen with the empty vector control (Figure 3D). Our data thus are consistent with the structural models of class I and class II complexes presented here.

### 2.4 RamA Mutations Affect Antibiotics Resistant Level in Clinical *K. pneumoniae* Strains

The clinical multidrug‐resistant isolate KP22 became susceptible to many antibiotics when ramA gene was knocked out (KP22Δ*ramA*) but multidrug resistance was rescued by RamA complementation (**Table** **1**). RamA‐complemented cells are particularly resistant to tigecycline, doxycycline, minocycline, and susceptibility of piperacillin‐tazobactam, ceftazidime, levofloxacin decreased significantly (Table 1). When RamA is mutated at H31 (H31D) so that interactions with *α*‐CTD are affected, the minimum inhibitory concentration (MIC) values of all antibiotics tested drop markedly and are the same as with KP22 Δ*ramA*, consistent with transcription data (Figure 3C,D). When mutating residues that affect *σ* ^70^ interactions, such as Y75R/D76R/D84R, the MIC only decreased slightly.

**Table 1:** Minimum inhibitory concentrations of KP22, KP22Δ*ramA and ramA* wild‐type/mutant complementation isolates against various antibiotics (S: susceptible; R: resistant; c *ramA*: wild‐type or mutant *ramA* complementation)

	KP22	KP22Δ*ramA*	KP22Δ*ramA*‐c *ramA*‐WT	KP22Δ*ramA*‐c *ramA‐* Y75R/D76R/D84R	KP22Δ*ramA*‐c *ramA‐* H31D	KP22Δ*ramA*‐c *ramA‐*H31D/Y75R/D76R/D84R
Piperacillin/tazobactam	16 S	≤4 S	16 S	8 S	≤4 S	≤4 S
Ceftazidime	0.5 S	≤0.12 S	0.5 S	0.25 S	≤0.12 S	≤0.12 S
Levofloxacin	1 S	≤0.12 S	1 S	1 S	≤0.12 S	≤0.12 S
Doxycycline	≥16 R	1 S	≥16 R	≥16 R	1 S	1 S
Minocycline	≥16 R	≤1 S	≥16 R	≥16 R	2 S	2 S
Tigecycline	≥8 R	≤0.5 S	≥8 R	≥8 R	≤0.5 S	≤0.5 S

## 3 Discussion

Many transcription activators regulate multiple genes and gene clusters. RamA, an activator in *K. pneumoniae*, regulates two sets of genes that play important roles in multidrug resistance. In this study, we confirm that RamA acts as a class I activator on one promoter and class II activator on another. Using cryoEM analysis of RamA class I and class II transcription complexes, supported by mutagenesis, we reveal how RamA is organized at the two promoters and how RamA interacts with RNAP and *σ* ^70^ to recruit/retain RNAP at the promoter site, thus enhancing transcription activities. RamA is the second example, apart from CAP/TAP, where structures of both class I and class II transcription complexes are available. Comparisons of these structures reveal both common and activator‐specific properties.

### 3.1 Structural Comparisons of RamA‐Class I Activator Complex with CAP‐Class I Activator Complex

**Figure 4:**
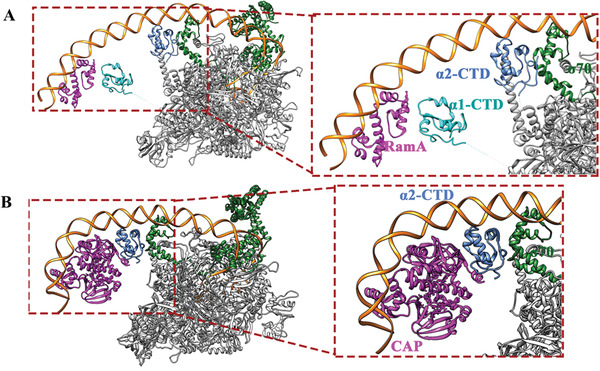
Comparisons of class I activator complexes. A) RamA‐class I complex, B) CAP‐class I complex, insets show enlarged views of activators interacting with *α*‐CTD and *σ* ^70^. RNAP core—gray, *σ* ^70^— dark green, *α*1‐CTD—cyan, *α*2‐CTD—light blue, activators—magenta.

In order to understand how different activators act on the different promoters, RamA‐class I activator complex structure was compared with that of CAP bound to *lac* promoter at 5.5 Å, the only other high/medium‐resolution structure available for a class I activator complex (**Figure** **4**A,B).^[^ ^16^ ^]^ CAP, which binds at ‐61.5 promoter DNA and contains a C‐terminal DNA‐binding domain and a N‐terminal c‐AMP‐binding‐domain, reaches downstream to interact with *α*‐CTD. The same *α*‐CTD contacts promoter DNA at ‐41 and interacts with *σ*R4 (Figure 4B), consistent with an earlier lower resolution structural model of CAP‐class I complex.^[^ ^16^, ^17^ ^]^ Although the interaction surface between *α*‐CTD and CAP is only ≈80 Å^2^ in the class I complex, there is an interaction network among CAP, *α*‐CTD, *σ*R4, and DNA. This interaction network connects CAP, *α*‐CTD, *σ* ^70^ and promoter DNA, allowing synergistic binding to the promoter DNA and contributing to its ability to act as a strong activator. RamA, on the other hand, only has one domain and binds to the promoter at ‐71, making it impossible to contact the *α*2‐CTD residing next to *σ*R4 (Figure 4A). Instead, *α*1‐CTD, which is not involved in promoter DNA‐binding, contacts RamA (Figure 4A). The interaction surfaces between RamA and *α*‐CTD is ≈60 Å^2^. Thus, in the Class I‐CAP complex, only a single *α*‐CTD is utilized to link CAP, DNA, and *σ*R4 together as an entity in binding to promoter, whereas in RamA complex, both *α*‐CTDs are utilized, with one interacting RamA, another one contacting DNA and *σ*R4, creating two separate binding sites. The different binding modes and different interaction surfaces contribute to the differences in their activation abilities.

The *α*2‐CTD that interacts with DNA and *σ*R4 is located similarly in CAP‐class I and RamA‐class I complexes. *α*2‐CTD is not visible in the absence of activators, suggesting that it is mobile and not localized. In the presence of activators, the *α*2‐CTD mobility could be confined, either by directly interacting with the activator as in CAP, or due to the *α*1‐CTD binding to the activator as in RamA, thus restricting the flexibility of their linker movements. Indeed, the two linkers are located not far from each other (Figure S7, Supporting Information), with *α*1‐CTD upstream of *α*2‐CTD. When *α*1‐CTD is restrained, such as when bound to RamA, the downstream *α*2‐CTD movement is restricted, allowing local concentration to increase thus engagement with DNA and *σ*R4.

### 3.2 Structural Comparisons of RamA‐Class II Complex with the CAP/TAP‐Class II Activator Complex

**Figure 5:**
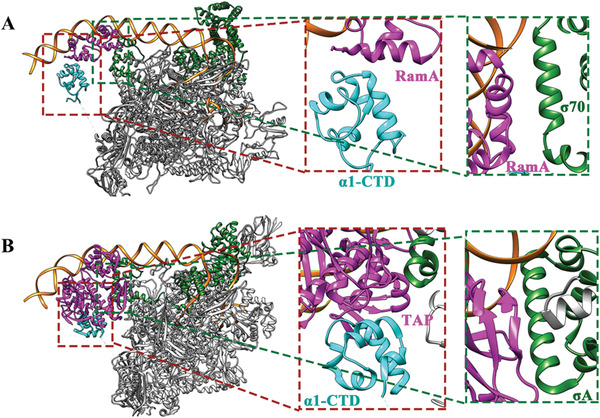
Comparisons of class II activator complexes. (A) RamA‐class II complex, B) TAP‐class II complex, Insets show enlarged views of activators interacting with *α*‐CTD and *σ* ^70^. RNAP core—gray, *σ* ^70^—dark green, *α*1‐CTD—cyan, activators—magenta.

Comparisons with the 4.4 Å crystal structure of TAP‐ promoter complex, and the recent 4.0–4.5 Å cryoEM structures of CAP–promoter complex at class II promoter sites, reveal similarities and differences.^[18,19]^ The structures of CAP–class II complexes reveal two conformations (state I and state II), with state II more similar to that of TAP‐class II complex.^[^ ^18^ ^]^ *α*‐CTD was not resolved in either of the CAP‐class II structures, while it is clearly visible in TAP‐class II complex as well as in RamA‐class II complex. Both TAP and RamA interact with *σ* ^70^ as well as *α*1‐CTD and RamA and TAP contact *σ*R4 at the same helix (**Figure** **5**A,B). Each monomer within the TAP dimer interacts with either *σ*R4 or *α*1‐CTD, while the RamA monomer instead uses the two HTH domains to contact *σ*R4 and *α*1‐CTD respectively (Figure 5A,B). The interaction surfaces between RamA and *α*‐CTD and between RamA and *σ*R4 are relatively small (≈60 Å^2^ and ≈100 Å^2^ respectively) and are mainly polar interactions in nature, as confirmed by the structural model. This is in stark contrast with that of CAP‐class II or TAP‐class II. The interaction surfaces between TAP and *α*‐CTD and between TAP and *σ*R4 in class II complex are larger at ≈430 Å^2^ and ≈130 Å^2^ respectively (Figure 5A,B).

Comparing with TAP, which forms extensive interactions with *α*1‐CTD/*σ* ^70^, the interaction surface between RamA and *α*1‐CTD/*σ* ^70^ is considerably smaller. Unlike in the TAP complex, where *α*1‐CTD sits on top of TAP and does not engage with DNA, *α*1‐CTD in the RamA complex contacts DNA, upstream of RamA. The CAP‐class II complexes reveal interactions between CAP and *σ*R4 as well as interactions with *β*’ subunit, the latter not observed in RamA‐class II complex.

### 3.3 Structural Basis of RamA‐Dependent Transcription Regulation

**Figure 6:**
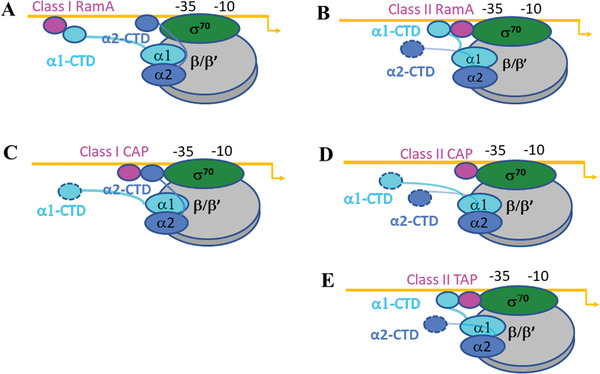
Structural models of class I and class II activators. *α*1 and *α*2 subunits are separated shaded. The orientation of the linkers connecting α‐CTD to α‐NTD restrict the relative locations of α‐CTDs along the promoter. The dashed outlines indicate that these *α*‐CTDs are flexible and not resolved in the structures

The interactions between *α*‐CTD of RNAP and RamA are maintained in both promoter complexes, although they are arranged in opposite directions relative to the promoter, thus one *α*‐CTD contacts RamA from upstream and another one from downstream (**Figure** **6**). These are made possible by the flexibility of the linker connecting *α*‐CTD to *α*‐NTD and due to RamA binding to opposing directions of the two promoters (Figure S9, Supporting Information). The RamA structural model consists of two HTH domains (H1‐H3 and H5‐H7) connected by a long central helix H4 (Figure S8C, Supporting Information). Based on the structure of MarA‐DNA complex, H3 and H6 interact with DNA major grooves (Figure S8C, Supporting Information). For MarA, H6 is shown to recognize 5’TTT while H3 is shown to recognize 5’CGTG located 7 bases apart (Figure S8B, Supporting Information). In *acrA* promoter, RamA binding site consists of similar sequences as in MarA binding site, 5’TTT‐7X‐CGTG but *micF* promoter contains 5’CACT‐7X ‐AAA (or in the complementary strand of 5’TTT‐7X‐AGTG) supporting the idea that RamA might bind to the two promoters in opposite directions (Figure S8B, Supporting Information). Differential orientations of marbox (MarA/SoxS/Rob binding site) have been identified in a number of MarA promoters,^[^ ^20^ ^]^ our results provide structural evidences to support this arrangement.

Disrupting the interface between RamA and RNAP‐*σ* ^70^ complex, the resistant level of several antibiotics in clinical *K. pneumoniae* strain decreased significantly. The results confirmed that RamA is involved in antibiotic resistance through transcription regulation. The MICs of tetracyclines and levofloxacin were maintained after disrupting the interaction between *σ* ^70^ and RamA. This might be because disrupting the interaction between *σ* ^70^ and RamA only partially influenced the transcription activation of class II promoter (*micF*). Meanwhile *micF* related porin deficiency is a relatively weak antimicrobial resistance mechanism in *K. pneumoniae*. Disrupting the interaction between *α*‐CTD and RamA impaired the transcription activation of class I promoter (*acrAB*) which is a major contributor to multidrug resistance and partial transcription activation of class II (*micF*) promoter. And the clinical multidrug‐resistant isolate KP22 restored susceptibility of doxycycline, minocycline, and tigecycline.

### 3.4 Activators Modulate Their Activities via a Diverse Range of Interactions between the Activator, *α*‐CTD and *σ* ^70^

In class I complex, RamA only interacts with *α*‐CTD and the interface is relatively small. Although the interaction surface between *α*‐CTD and CAP is only ≈80 Å^2^ in the CAP‐class I complex, the cooperative effects of the interaction network among CAP, *α*‐CTD, *σ*R4, and DNA enhance the overall affinity. In class II complex, the interaction surfaces between RamA and *α*‐CTD and between RamA and *σ*R4 are relatively small. This is in stark contrast with that of TAP‐class II. The results are reflected in their different abilities in activation, with two to fourfolds for RamA and comparable with MarA^[^ ^21^ ^]^ but considerably lower than that of CAP which is shown to increase transcription by four to eight fold for class I promoters^[^ ^22^ ^]^ and up to 26‐fold on class II promoters.^[^ ^23^ ^]^

Thus, activators can use different interactions with *α*‐CTD and *σ* ^70^ to alter the strength of activation as a strategy to regulate the activator–promoter relationship. These features made RamA a weaker transcription activator on class I and class II promoter compared with CAP/TAP.

### 3.5 Common Features in Activator Binding

Structural comparisons between RamA and CAP/TAP complexes reveal some common features that could be applicable to other activators. First, *σ*R4 is involved in stabilizing all complexes, either through binding to *α*2‐CTD as in class I complexes or directly interacting with the activators as in class II activator complexes (Figure 6). Second, the two *α*‐CTDs, although flexible, seem to show particularly preferred locations. Consequently, the two *α*‐CTDs have overlapping but distinct roles. *α*1‐CTD seems to be the main activator‐interacting *α*‐CTD while *α*2‐CTD is involved in engaging with *σ*R4. Third, *α*‐CTDs are flexibily utilized in coordinating a diverse range of activators bound at different locations. For example, either one or both *α*‐CTDs could be utilized depending on where the activator binds and the size of the activator protein, as seen with RamA and CAP in class I promoters. Fourthly, *α*‐CTD could engage with a range of cofactors such as different activators, DNA, and *σ*R4 through its variable surfaces (Figure S10, Supporting Information). This is due to the highly variable surface charge distribution of *α*‐CTD (Figure S10, Supporting Information). Through altering the type and number of engagements between *α*‐CTD and DNA, activator and *σ*R4 as well as either one or two *α*‐CTDs, the system could fine tune to accommodate a wide range of activator‐DNA engagements.

## 4 Experimental Section

### Bacterial Strains and Plasmids

Bacterial strains and plasmids used in this study are listed in Table S1 (Supporting Information). Antibiotics were used at the following concentrations: ampicillin (Amp) at 50 µg mL^‐1^, chloramphenicol (Cm) at 34 µg mL^‐1^. *E. coli* strains were grown in Luria‐Bertani (LB) medium at 37 °C.

### Electroporation

*E. coli* MG1655 and *K. pneumoniae* KP22ΔRamA strains were prepared for electroporation. Cells grew to exponential phases in LB medium were washed twice and resuspended in 50 µL of sterile ddH_2_O and mixed with vectors. Electroporation was performed in a 1 mm Micropulser cuvette (Bio‐Rad) at 1800 V. Cells were recovered in LB medium at 37 °C for 1 h before plating onto selective LB agar plates and incubated for overnight at 37 °C.

### Mutagenesis of RamA

Mutagenesis was performed by inverse PCR and Infusion clone.^[^ ^24^ ^]^ pOPINF‐RamA, pBAD‐18cm‐RamA, and pHSG398‐RamA plasmids were amplified by using a pair of 15 bp overlapped primers which incorporated the desired mutation using CloneAmp HiFi PCR Premix (Takara) kit. The In‐Fusion (Takara) reaction was performed by using the inverse PCR products. The reactions were transformed into *E. coli* 10*β* competent cells (NEB), spread on selective antibiotics plates, and confirmed by sequencing.

### Protein Purification

RamA wild‐type and its mutants with a N‐terminal polyhistidine tag were expressed in *E. coli* BL21(DE3), and were purified as described for the homologous protein MarA. Briefly, the cells were disrupted by sonication and harvested via centrifugation. The supernatant fluid was discarded and the pellet was rinsed with 2% Trinton X‐100, 20 × 10^‐3^ m Tris and 0.5 m NaCl (pH 7.5) for three times. Finally, the pellet was resuspended in 40 mL of 8 m urea buffer and centrifuged at 18000 × *g* for 50 min. The supernatant was collected and further purified through Ni affinity chromatography (GE Healthcare). Then, denatured RamA protein was collected and refolded by dialysis at 4 ℃ overnight in buffer 20% (vol/vol) glycerol, 20 × 10^‐3^ m Tris (pH 8.0), 5 × 10^‐3^ m TCEP and 0.5 m NaCl. The purified protein was frozen directly without concentration after dialysis (Figure S11, Supporting Information).

*E. coli* RNAP and *σ* ^70^ proteins were expressed and purified as described.^[^ ^25^ ^]^ The identity of RNAP sequences between *E. coli and K. pneumoniae* is 100%. The identity of *σ* ^70^ is 96% while R4 region is completely conserved (100% identity). The Plasmid pGEMABC encoding *E. coli rpoA*, *rpoB*, and *rpoC* and plasmid pACYCDuet‐omega (encoding *rpoZ*) were coexpressed in BL21(DE3) cells. Cells were harvested via centrifugation and re‐suspended in lysis buffer (50 × 10^‐3^ m Tris‐HCl (pH 8.0), 1 × 10^‐3^ m EDTA, 5 × 10^‐3^ m DTT) supplemented with 1 × 10^‐3^ m PMSF and protease inhibitor tablet (Sigma) and lysed through sonication. The soluble supernatant was collected and precipitated with 0.4% (final concentration) Polymin P (pH 7.9), followed by centrifugation. The precipitate was then washed and eluted with 1 m NaCl and precipitated with ammonium sulfate. The precipitates were suspended and dialyzed in 100 × 10^‐3^ m NaCl buffer. The sample was further purified with BioRex70 (BioRad) and HiTrap Q column (GE Healthcare). *σ* ^70^ from *E. coli* was expressed in BL21 (DE3) cells. Cells were harvested through centrifugation and lysed by sonication. The soluble supernatant was collected and further purified through Ni and Heparin affinity chromatography (GE Healthcare). *σ* ^70^ holoenzyme was formed by incubating RNAP with a four‐fold excess of *σ* ^70^ for 30 min at 4 °C before size exclusion chromatography using a Superose 6 10/300 column (GE Healthcare) equilibrated in buffer GF (10 × 10^‐3^ m Tris‐HCl pH 8.0, 150 × 10^‐3^ m NaCl, 10 × 10^‐3^ m MgCl_2_ and 5% glycerol) (Figure S11, Supporting Information).

### Activator Complex Formation

The transcription start site, ‐10 and ‐35 binding sites of *acrA* and *micF* promoters were derived based on the sequence conservation between *K. pneumoniae and E. coli*, with the latter annotated in the EcoCYC database (ecocyc.org). The RamA binding sites on these two promoters are identified according to the consensus promoter binding sequences of RamA, MarA, and SoxS: TTTXXXXXXXCGTG. The promoter DNA sequences containing RamA binding site, RNAP‐*σ* ^70^ binding sites used in this study are shown in Figure 1A.

To form the class I RamA‐promoter‐holoenzyme complex, a synthetic DNA scaffold corresponding to the *acrA* promoter region between positions ‐85 and +15 relative to the expected transcription start site was designed. The synthetic promoter, which contains RamA binding region, *α*‐CTD binding site, the consensus ‐35 and ‐10 hexamers, were prepared by annealing the nontemplate (NT) strand to an equal molar amount of the template strand DNA that is complementary to the NT‐strand except for a 6‐nucleotide (nt) discriminator region. The class I promoter complex was assembled by directly incubating the *σ* ^70^‐RNAP holoenzyme with a fivefold molar excess of the pre‐formed DNA–RamA complex in assembly buffer (20 × 10^‐3^ m Tris pH 7.5, 50 × 10^‐3^ m NaCl, 0.1 × 10^‐3^ m EDTA, 5 × 10^‐3^ m MgCl_2_, 10% glycerol) at 4 °C for 30 min and following at 37 °C for 10 min in the presence of GTP, ATP, and CTP (2 × 10^‐3^ m each). The complex was further purified by gel filtration chromatography using a Superose 6 10/300 column (GE Healthcare) with buffer EM (20 × 10^‐3^ m Tris pH 8, 50 × 10^‐3^ m NaCl) to remove the extra RamA‐DNA complex.

The class II RamA‐promoter‐holoenzyme complex was formed in the same way as class I complex except for the synthetic DNA scaffold corresponding to the *micF* promoter region between positions ‐57 and +16 relative to the transcription start site. CTP and GTP were used in the complex assembly. The promoter sequences used are shown in Figure 1.

### Cryo‐EM Sample Preparation

The electron microscopy grids were cleaned by argon oxygen for 45 s. A droplet of 4 µL of freshly purified samples containing class I complexes were applied at a concentration of 0.5 mg mL^‐1^ to R2/2 holey carbon grids (Quantifoil) whereas the class II complex at a concentration of 0.5 mg mL^‐1^ were applied to R1.2/1.3 holey grids (Quantifoil), blotted 0.5 s with the blotting force ‐9, and vitrified using a Vitrobot Mark IV (FEI) at 4 °C and 100% humidity. The grids were then flash frozen in liquid ethane and stored in the liquid nitrogen before data collection.

### Cryo‐Electron Microscopy Data Collection

For class I complex, the cryo‐EM data were collected at eBIC (Diamond Light Source, UK) on a Titan Krios using EPU (Thermo Fisher Scientific) operated at 300 kV and a Falcon III direct electron detector (Thermo Fisher Scientific). The data were collected with a defocus (underfocus) range between ‐1.8 to ‐3.6 µm. A total of 3472 micrographs were collected at a pixel size of 1.085 Å per pixel and a dose of 55 e^–^ Å^‐2^ and each micrograph was fractionated into 39 frames (1.42 e^–^/Å^2^/frame). Similarly for class II complex, the data were collected with a defocus range between ‐1.5 and ‐3 µm. A total of 2878 micrographs were collected at pixel size of 1.084 Å per pixel and a dose of 79.1 e^–^ Å^‐2^ and each micrograph was fractioned into 39 frames (1.73 e^–^/Å^2^/frame).

### Image Processing

Image processing procedures for all the datasets are summarized in Figure S1 (Supporting Information). Frame alignment and dose weighting were carried out by MotionCor2^[^ ^26^ ^]^ before estimating CTF parameters using Gctf^[^ ^19^ ^]^ and particle picking with Gautomatch (https://www.mrc‐lmb.cam.ac.uk/kzhang/Gautomatch/). Picked particles were extracted into boxes of 368 × 368 pixels for class I complex and 288 × 288 pixels for class II complex. Initial 2D classification was carried out in Cryosparc^[^ ^27^ ^]^ in order to remove junk particles. All further processing was performed in RELION 3.0.^[^ ^28^ ^]^ After further rounds of 2D classifications to clean up datasets, 3D classification was then performed on 638469 particles for class I complex and 512167 particles for class II complex, using a 60 Å low pass filtered reference model generated from a *E. coli* transcription initiation complex structure (PDB ID 4YLP) with C1 symmetry. For the class I complex, one out of six classes which showed clear extra density upstream from RNAP‐*σ* ^70^, suggestive of having RamA bound, was selected for 3D autorefinement, followed by postprocessing, yielding a reconstruction with 4.1 Å global resolution. However, density quality around RamA region is poor. Particles were then subjected to further rounds of 3D classification using a soft mask focused on the RamA. Despite multiple rounds of focused classification followed by multibody refinement surrounding the RamA‐DNA around the upstream DNA and the rest of the complex as two separate bodies, the density around RamA‐DNA remain poor (12.1 Å reconstruction) (Figures S1 and S3, Supporting Information). Similar processing strategies were followed for the class II complex. The overall reconstruction is 4Å and the region surrounding RamA part around 6 Å for RamA part (Figures S2 and S4, Supporting Information).

### Structure Modeling and Refinement

The structure of transcription open complex (PDB code:4YLP) was used as an initial model for the model building of both RamA‐class I and RamA‐class II reconstructions. Briefly, the model 4YLP was first fitted into the RamA‐class I and RamA‐class II density map, respectively, in Chimera.^[^ ^29^ ^]^ The predicted RamA structural model was manually fitted into the extra density of the RamA‐class I and RamA‐class II map in Coot.^[^ ^30^ ^]^ Jelly body refinement in Refmac^[^ ^31^ ^]^ and real‐space refinement in Phenix^[^ ^32^ ^]^ were used to improve the model quality. The final statistics of the models are in **Table** **2**. The figures used for structure analysis and comparison were produced in Pymol (The PyMOL Molecular Graphics System, Version 2.0 Schrödinger, LLC) and UCSF Chimera.^[^ ^29^ ^]^

**Table 2:** Cryo‐EM data collection and structural refinement statistics for class I and class II complexes

	Class I	Class II
Data collection and processing		
Magnification Total micrographs Exposure time [s] Movie frames Pixel size [Å]	75 000 3472 1 39 1.085	75 000 2878 1 39 1.084
Defocus range [µm]	‐1.8 to ‐3.6	‐1.5 to ‐3
Voltage [kV]	300	300
Electron dose [e^–^ Å^–2^] Detector Total particles FSC threshold Reconstruction Software Symmetry Particles Resolution [Å]	55 Falcon III 1328947 0.143 Relion C1 39703 4.2	79.1 Falcon III 774955 0.143 Relion C1 74282 4.5
Refinement Resolution [Å] R.m.s.deviations	4.2	4.5
Bond length [Å]	0.003	0.003
Bond angle [°]	0.772	0.765
Ramachandran plot		
Favored regions [%]	92.71	94.73
Allowed regions [%]	6.97	5.07
Outlier Validation All‐atom clash score Rotamer outliers (%) C‐beta deviations	0.33 4.48 0.29 0	0.20 5.05 0.21 0

### Short Primed (sp) RNA Assay

In a 10 *μ*L reaction, 100 × 10^‐9^ m holoenzyme, 400 × 10^‐9^ m promoter DNA (same as those used in cryoEM analysis) and 400 × 10^‐9^ m RamA were mixed and incubated in assembly buffer for 30 min on ice. Synthesis of spRNA was initiated with an elongation mix containing 100 µg mL^‐1^ heparin, 0.5 × 10^‐3^ m of GpA for *acrA* promoter and CpG for *micF* promoter, and 0.2 mCi mL^‐1^ [*α* ^32^P] CTP (3000 Ci mmol^‐1^) and left for 10 min at 37 °C. Reactions were quenched with loading dye and analyzed on 20% denaturing gels. All the reactions had three replicates. Transcripts were visualized and quantified using a Fuji FLA‐5000 Phosphorimager and ImageJ software.

### *β‐*Galactosidase Assays

*β*‐galactosidase (LacZ) assays were used to quantify the level of LacZ protein production and thus activity of wild‐type and mutant (H31D, Y75R/D76R/D84R and H31D/Y75R/D76R/D84R) RamA. pBAD18‐CmR, pBAD18‐CmR‐RamA wildtype, and mutant vectors were transformed into MG1566 cells (not expressing RamA) containing P*acrA–lacZ* fusion or P*micF‐ lacZ* fusion plasmid. Cells were grown overnight at 37 °C in LB. Then diluted 100‐fold in the same medium containing 0.2% arabinose and left for growth for 24 h. The β‐galactosidase (β‐gal) assay was performed at three timepoints (6h, 12h and 24h) as described.^[^ ^33^ ^]^ All experiments were repeated three times and the results were used to calculate the averages that were then plotted in the graphs.

### Antimicrobial Susceptibility Testing

The lab has knocked out *ramA* gene from a clinical multiple drug resistant *K. pneumoniae* strain, KP22ΔRamA.^[^ ^3^ ^]^ To confirm whether RamA mutants would influence the antibiotic resistance in clinical strains, the MICs of several antibiotics were tested for RamA wild‐type and mutant complementation in KP22ΔRamA.

pHSG398, pHSG398‐RamA wildtype, and mutant vectors were transformed into KP22ΔRamA cells. Antimicrobial susceptibility test was performed using broth microdilution methods based on Clinical and Laboratory Standards Institute (CLSI), 2019 guidelines.^[^ ^34^ ^]^ The MICs of piperacillin‐tazobactam, ceftazidime, levofloxacin, tigecycline, doxycycline, and minocycline were determined according to CLSI standards.

### Statistical Analysis

Two‐way analysis of variance (ANOVA) as implemented by GraphPad PRISM 6 was used for data analysis of *β*‐galactosidase assay and P values smaller than 0.05 were considered statistically significant.

## Conflict of Interest

The authors declare no conflict of interest.

## Author Contributions

M.H. and F.Y. contributed equally to this work. X.Z., M.W., and M.B. designed the studies. M.H. and F.Y. prepared the samples and performed the cryoEM analysis. F.Y. built and refined the structural models. M.H., M.J., I.K.‐L., Q.X., and X.Q. conducted the biochemical experiments. X.Z. wrote the manuscript with input from all the authors.

## Supporting information

Supporting Information

## Data Availability

The cryoEM maps and coordinates of the RamA class I and class II complexes are deposited and available at PDB and EMDB with access codes of EMD‐12157, EMD‐12156 and PDB ID 7BEG and 7BEF respectively.
