# Supplementary material for: Structures of Class I and Class II Transcription Complexes Reveal the Molecular Basis of RamA‐Dependent Transcription Activation
Source: Adv Sci (Weinh). 2021 Nov 10;9(4):2103669. doi: 10.1002/advs.202103669 (PMC8811837; doi:10.1002/advs.202103669)
Supplement: Supplementary file 1 — Supporting Information [file ADVS-9-2103669-s001.pdf]

ADVANCED SCIENCE
Open Access

Supporting Information

for *Adv. Sci.*, DOI: 10.1002/advs.202103669

Structures of Class I and Class II Transcription Complexes Reveal the Molecular Basis of RamAdependent Transcription Activation

Min Hao,[#] Fuzhou Ye,[#] Milija Jovanovic, Ioly Kotta-Loizou, Qingqing Xu, Xiaohua Qin, Martin Buck, Xiaodong Zhang, and Minggui Wang,**

Supplementary Materials for

Structures of class I and class II transcription complexes reveal the molecular basis of RamA-dependent transcription activation

This PDF file includes:

Figs. S1 to S11
Tables S1

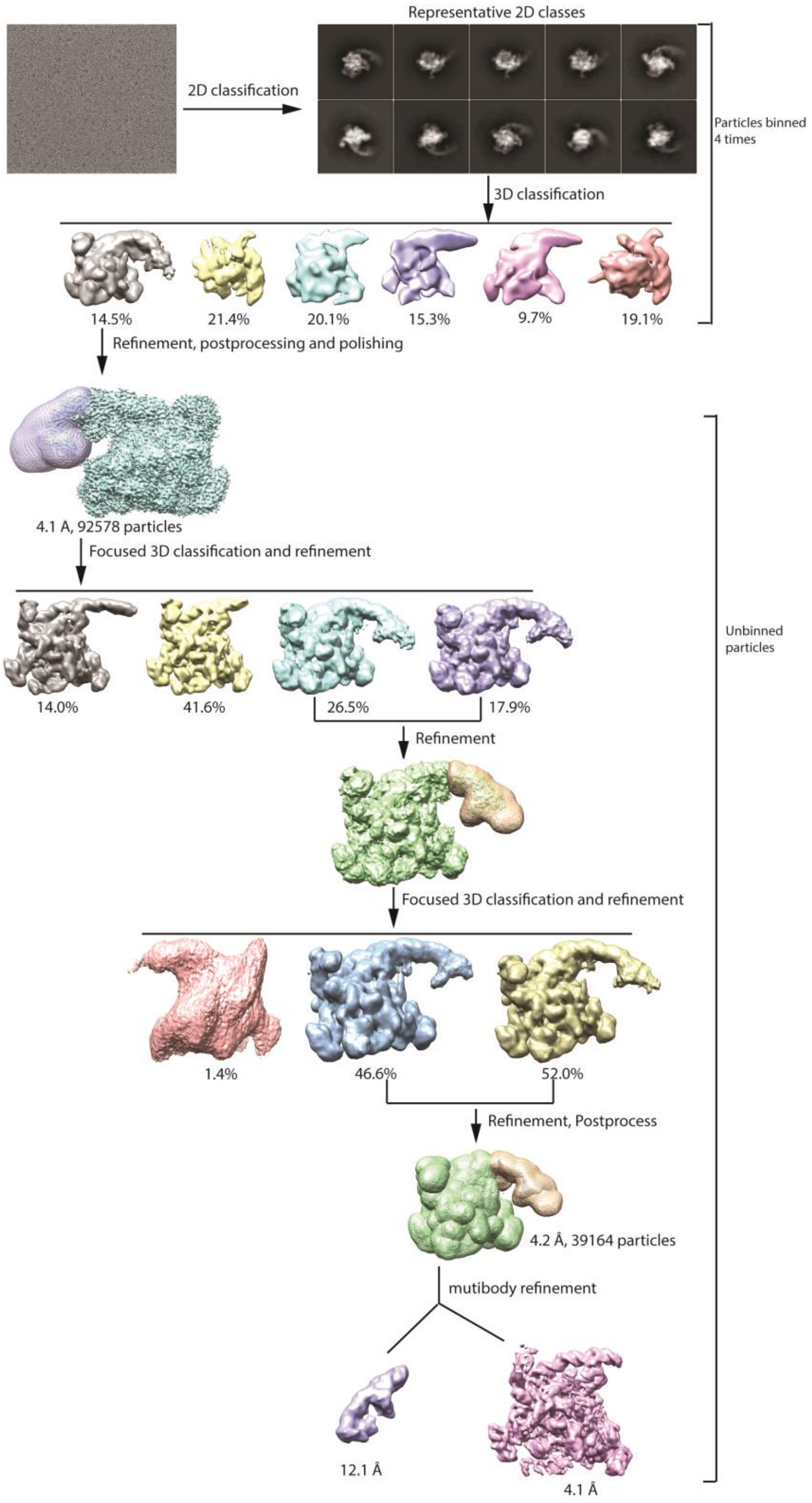

Fig. S1. Cryo-EM micrograph, 2D classification and data processing flow chart of class I complex.

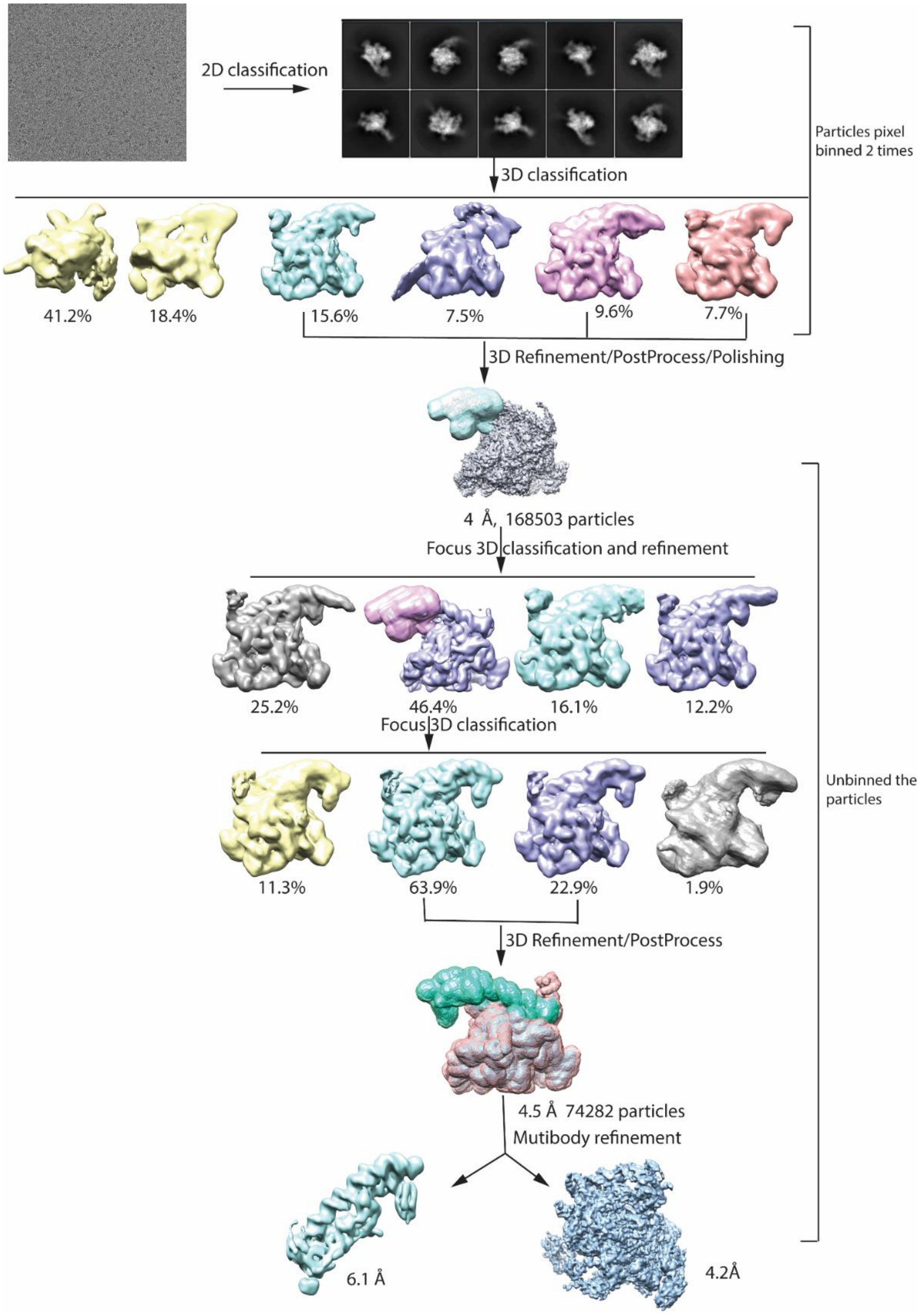

Fig. S2. Cryo-EM micrograph, 2D classification and data processing flow chart of class 2 complex.

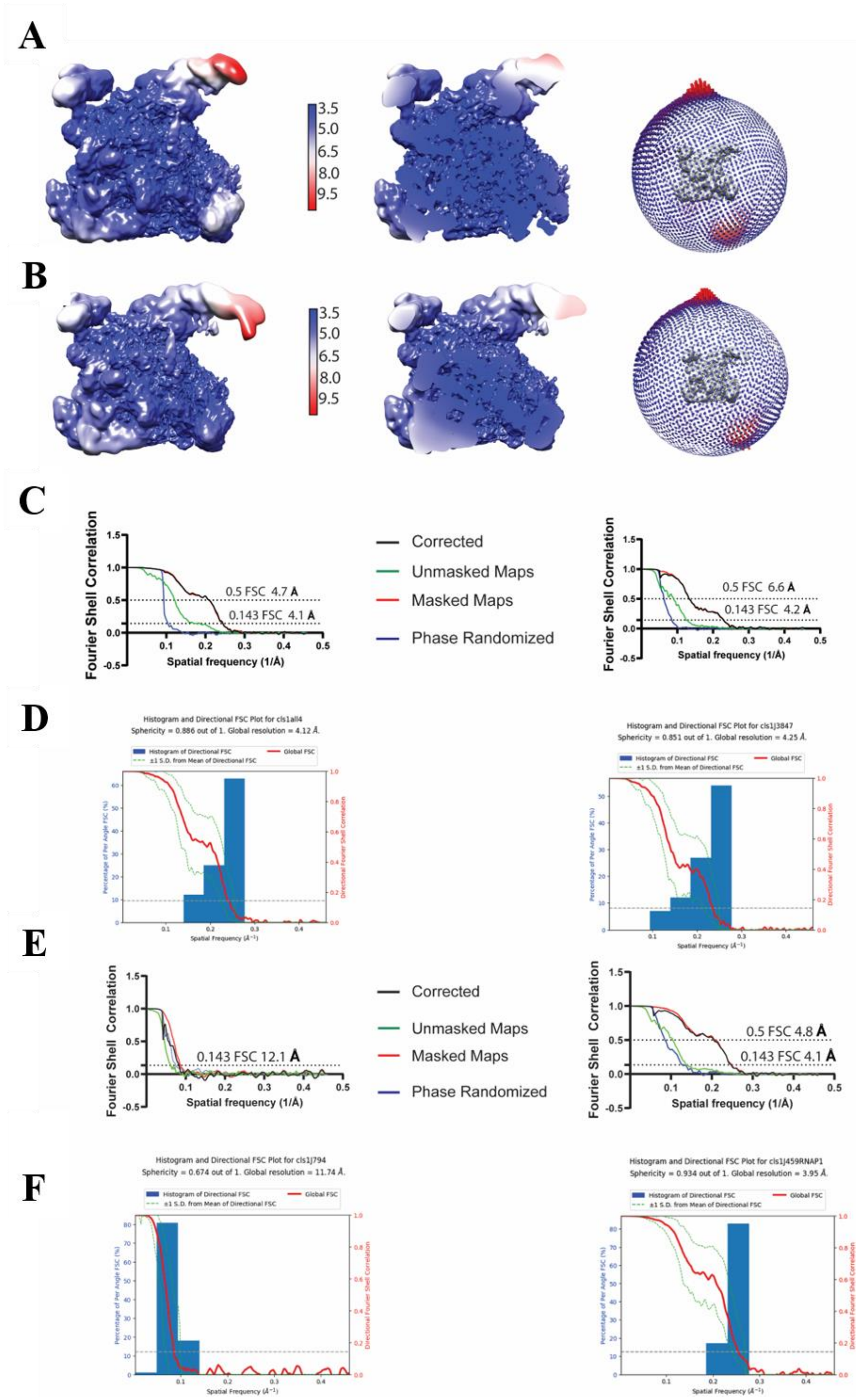

Fig. S3. Image reconstruction qualities of class I complex as judged by local resolution maps, angular distribution and FSC. A) – local resolution map and angular distribution of particles of the reconstruction after initial 3D classification and refinement B) same as A) for the reconstruction after further rounds of focused 3D classifications around the RamA. C-D) FSC curves, histograms and directional FSC plots for the two reconstructions. E-F) FSC curves, histograms and directional FSC plots for the two multi-bodies defined in Figure S1.

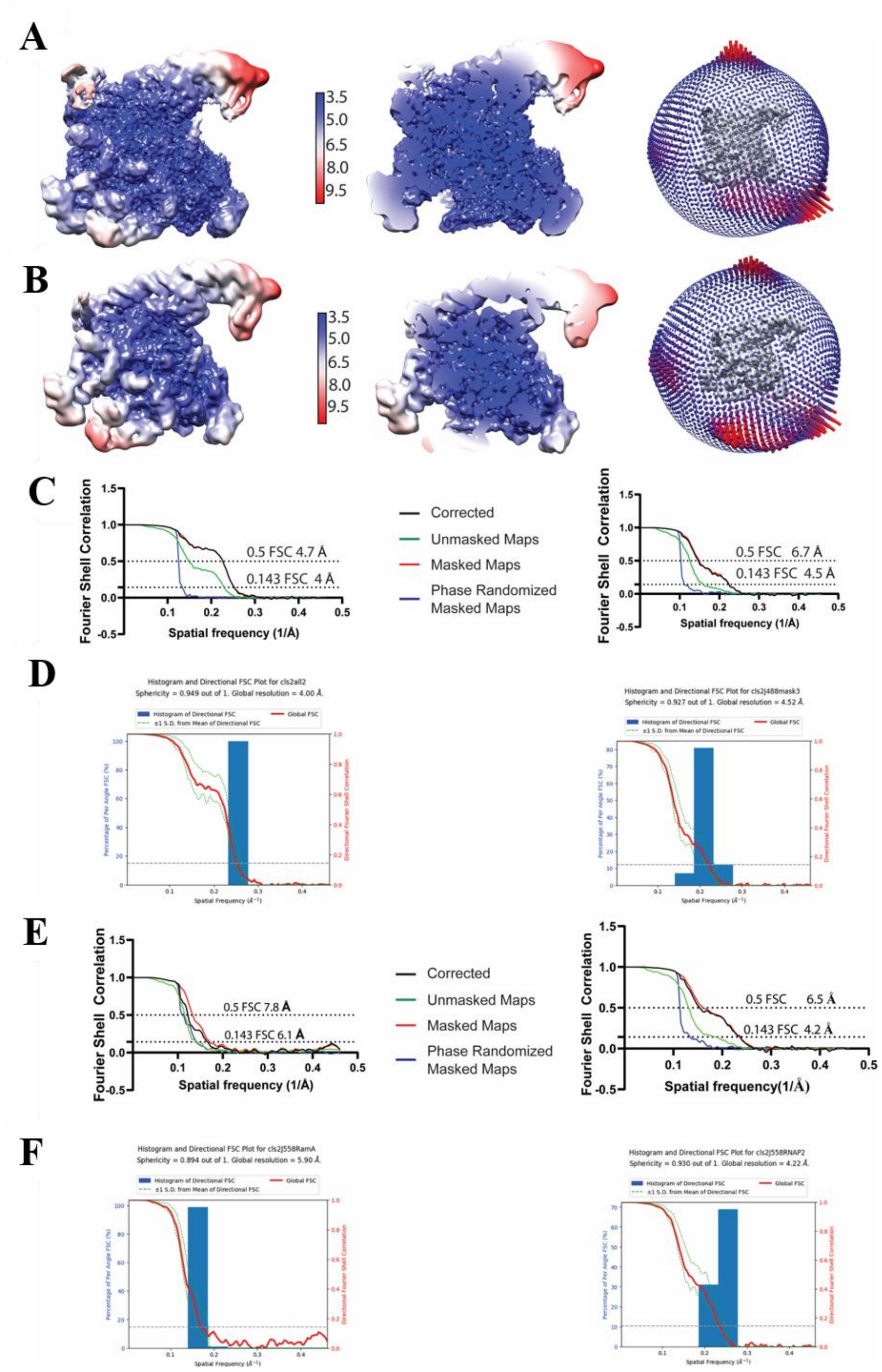

Fig. S4. Image reconstruction qualities of class II complex as judged by local resolution maps, angular distribution and FSC. A) – local resolution map and angular distribution of particles of the reconstruction after initial 3D classification and refinement B) same as A) for the reconstruction after further rounds of focused 3D classifications around the RamA. C-D) FSC curves, histograms and directional FSC plots for the two reconstructions. E-F) FSC curves, histograms and directional FSC plots for the two multi-bodies defined in Figure S2.

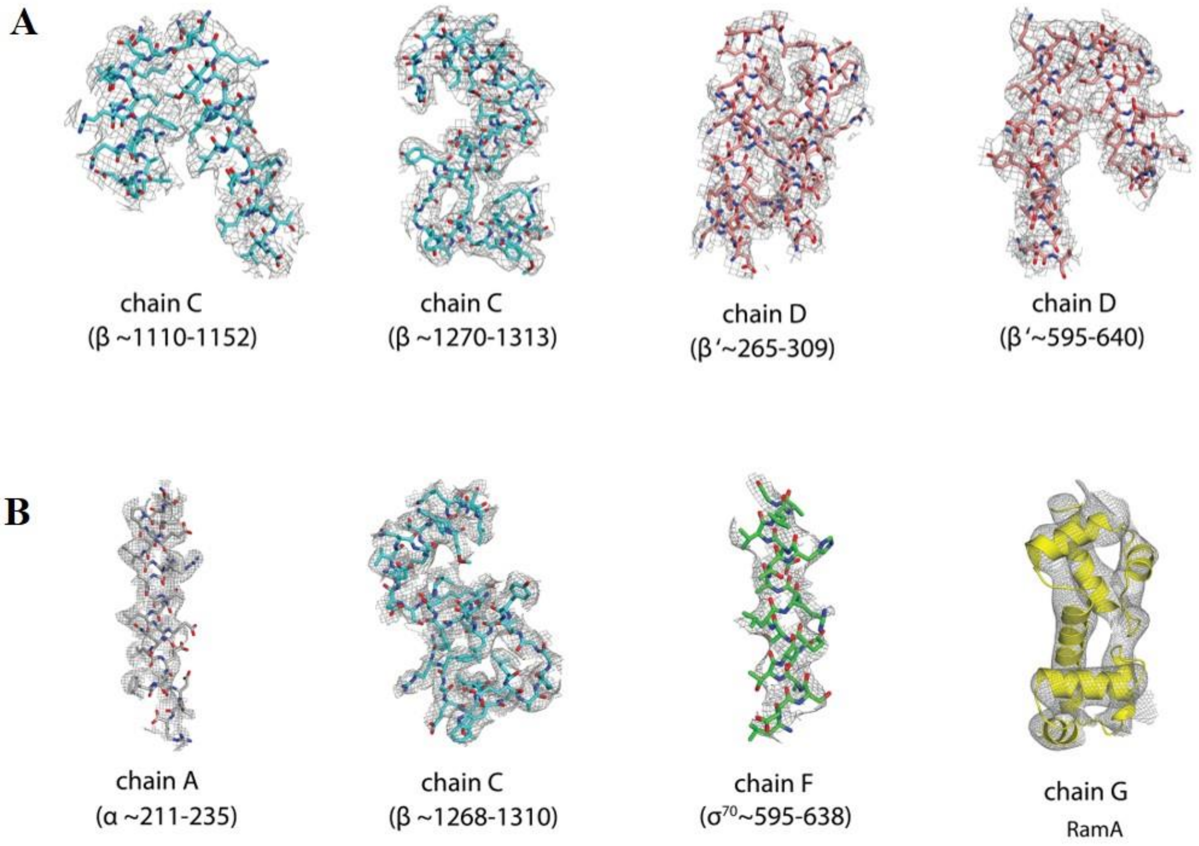

Fig. S5. Representative maps of the reconstruction including regions of RNAP-σ^{70} and RamA. A) class I complex; B) class II complex.

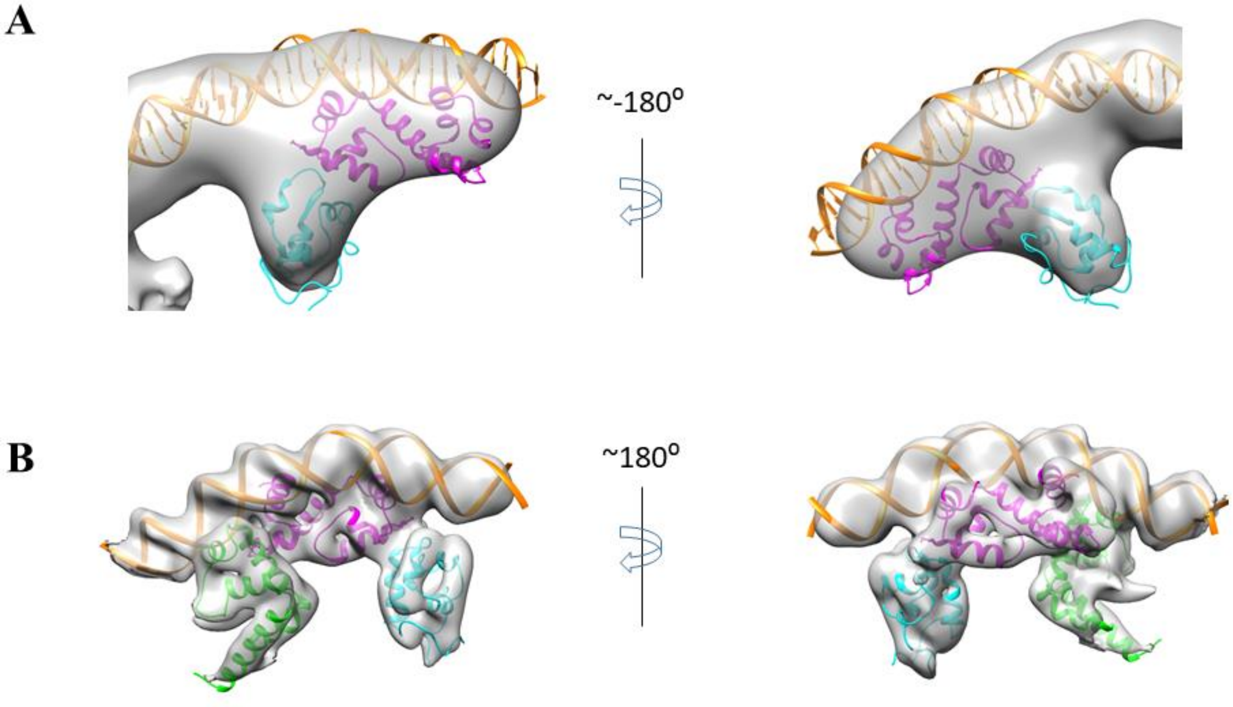

Fig. S6. Enlarged region of Cryo-EM reconstructions. A) RamA-αCTD part from RamA Class I complex. B) RamA-αCTD-σ^{70} part from RamA Class II complex.

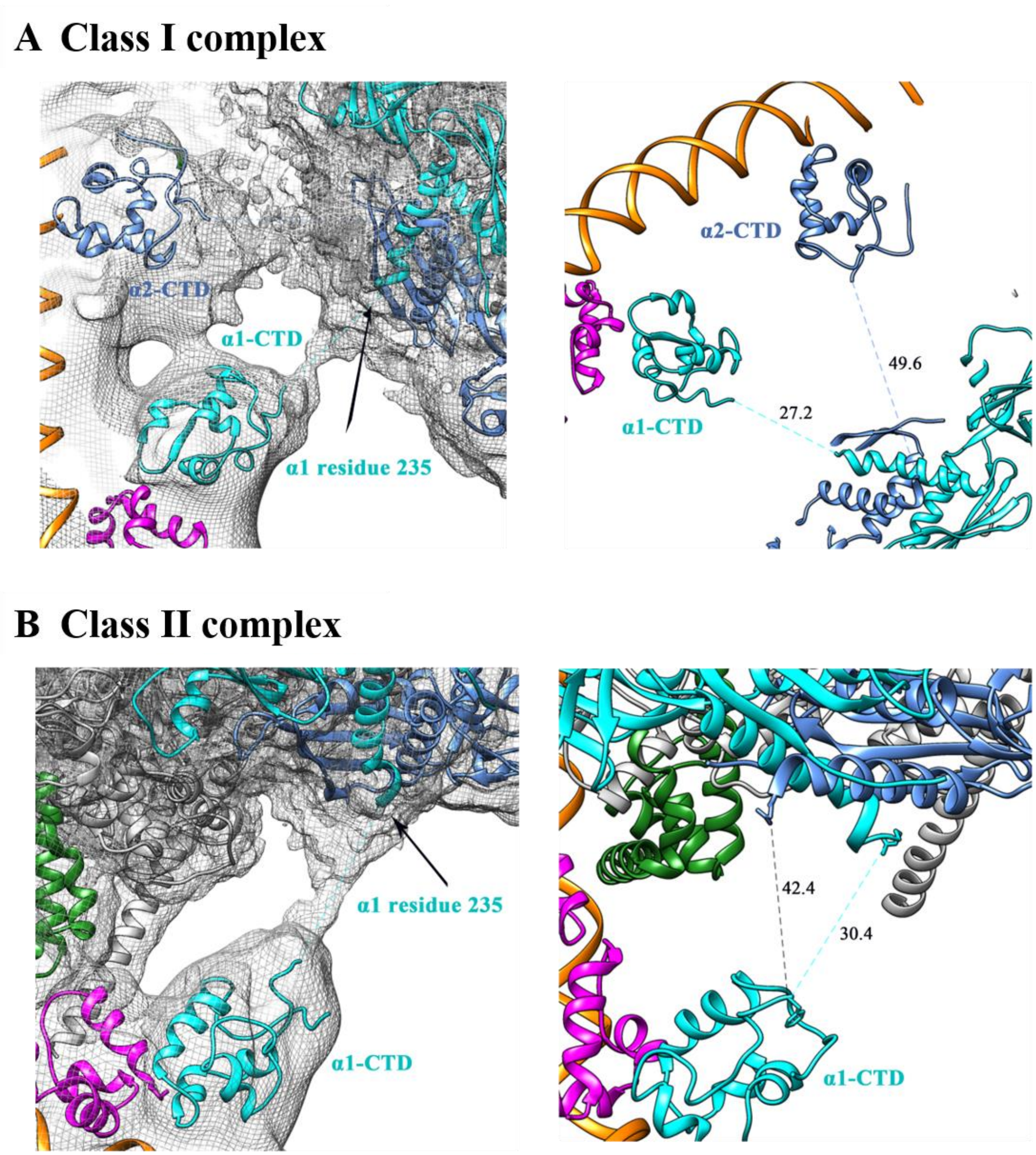

Fig. S7. RamA-interacting α-CTD is connected to a1 in both class I and class II activator complexes. A) class I activator complex. Left panel: lowering the threshold shows clear electron density (grey mesh) connecting α1-CTD interacts with RamA. Arrangement of α-NTD dimers dictate that α2-CTD is restricted to interact with promoter DNA close to core promoter regions as in class I activator complex. B) Class II activator complex lowering threshold of electron density (grey mesh) shows α1-CTD interacts with RamA. Thus α1-CTD is involved in activator binding for both class I and class II activators.

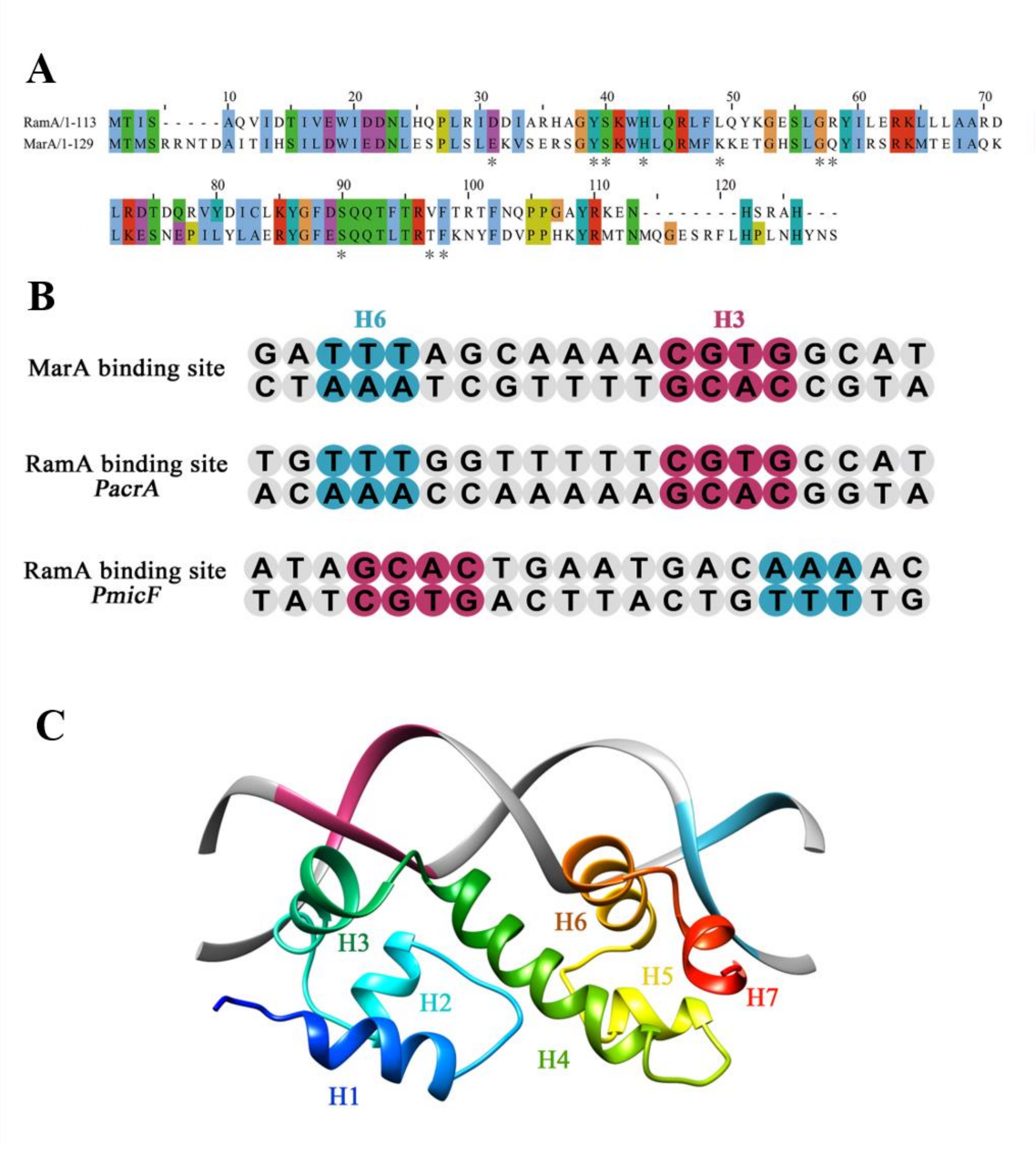

Fig. S8. RamA structural model and its interactions with promoter DNA. A). Sequence alignment of RamA with MarA of which a structural model is generated from. B) DNA sequences recognized by MarA and corresponding RamA binding sites of *acrA* and *micF* promoters. C) Predicted RamA-DNA model for class I and class II promoters, AAA and GCAC sequences are highlighted. RamA is colored in rainbow with N-terminus in blue and C-terminus in red.

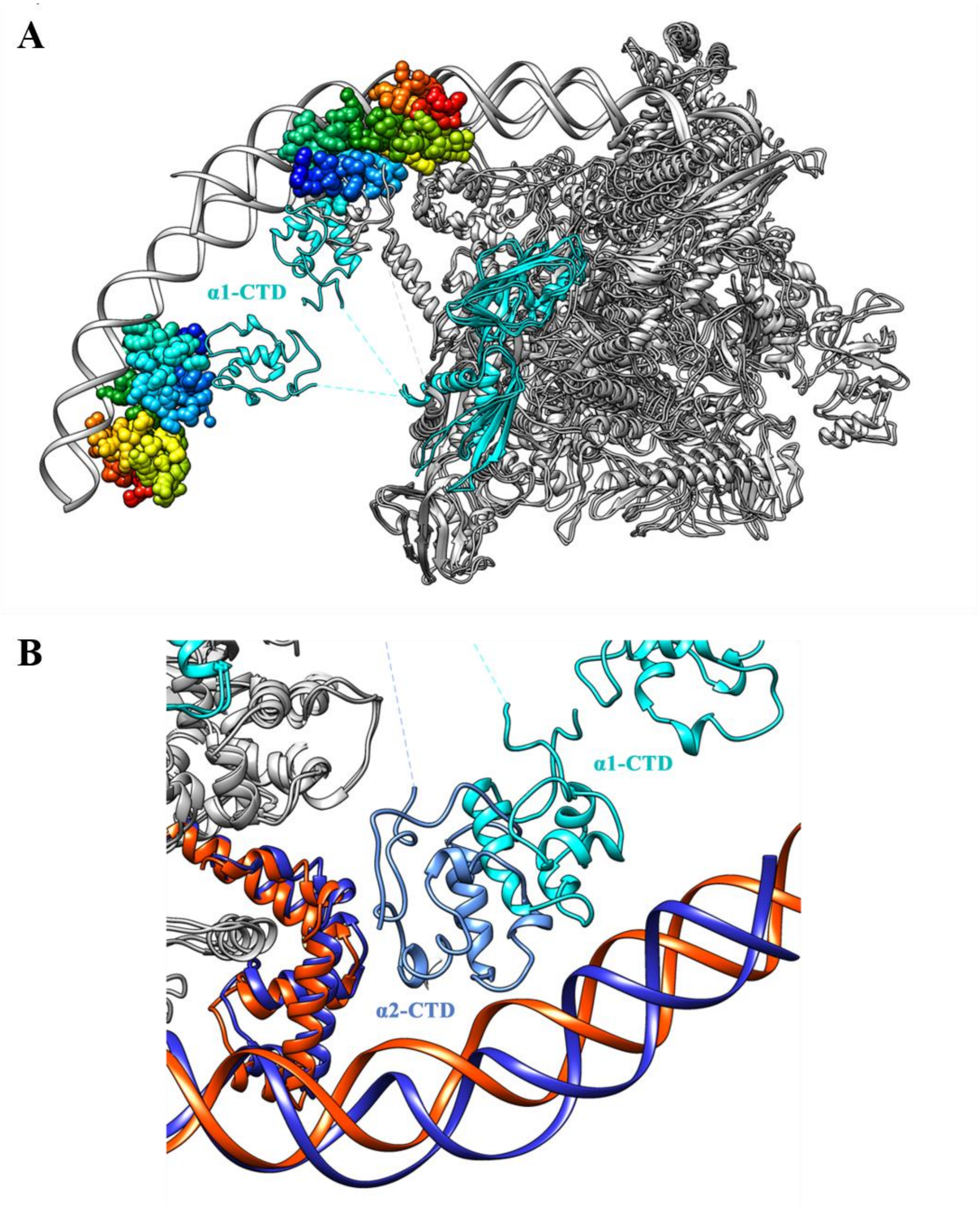

Fig. S9. Comparison of RamA as class I and class II activators. A) The two complexes overlaid on RNAP (grey ribbons) showing the different locations and orientations of RamA (spheres). RamA is colored in rainbow with N-terminus in blue and C-terminus in Red. B) Zoomed in view around α-CTD and σR4 and promoter DNAs. α1-CTD: cyan, α2-CTD: light blue. Blue – class II, red – class I.

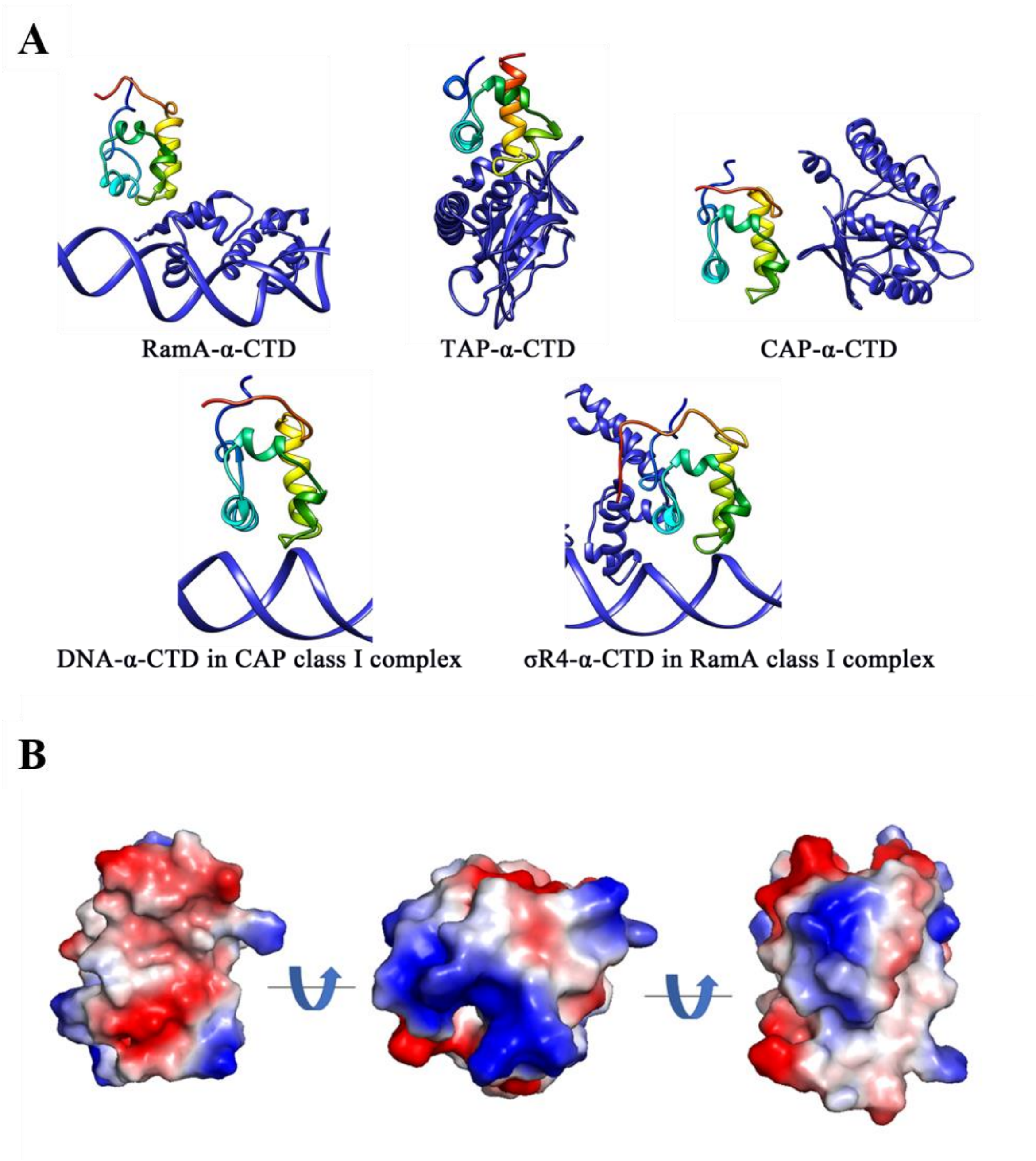

Fig. S10. A) diverse range of interactions between α-CTD and DNA, activator and σR4. α-CTD is colored in rainbow with N-terminus blue and C-terminus read. Other interacting partners are colored blue. B) charge distribution of α-CTD in three views. Blue – positively charged, red – negatively charged, white – neutrally charged.

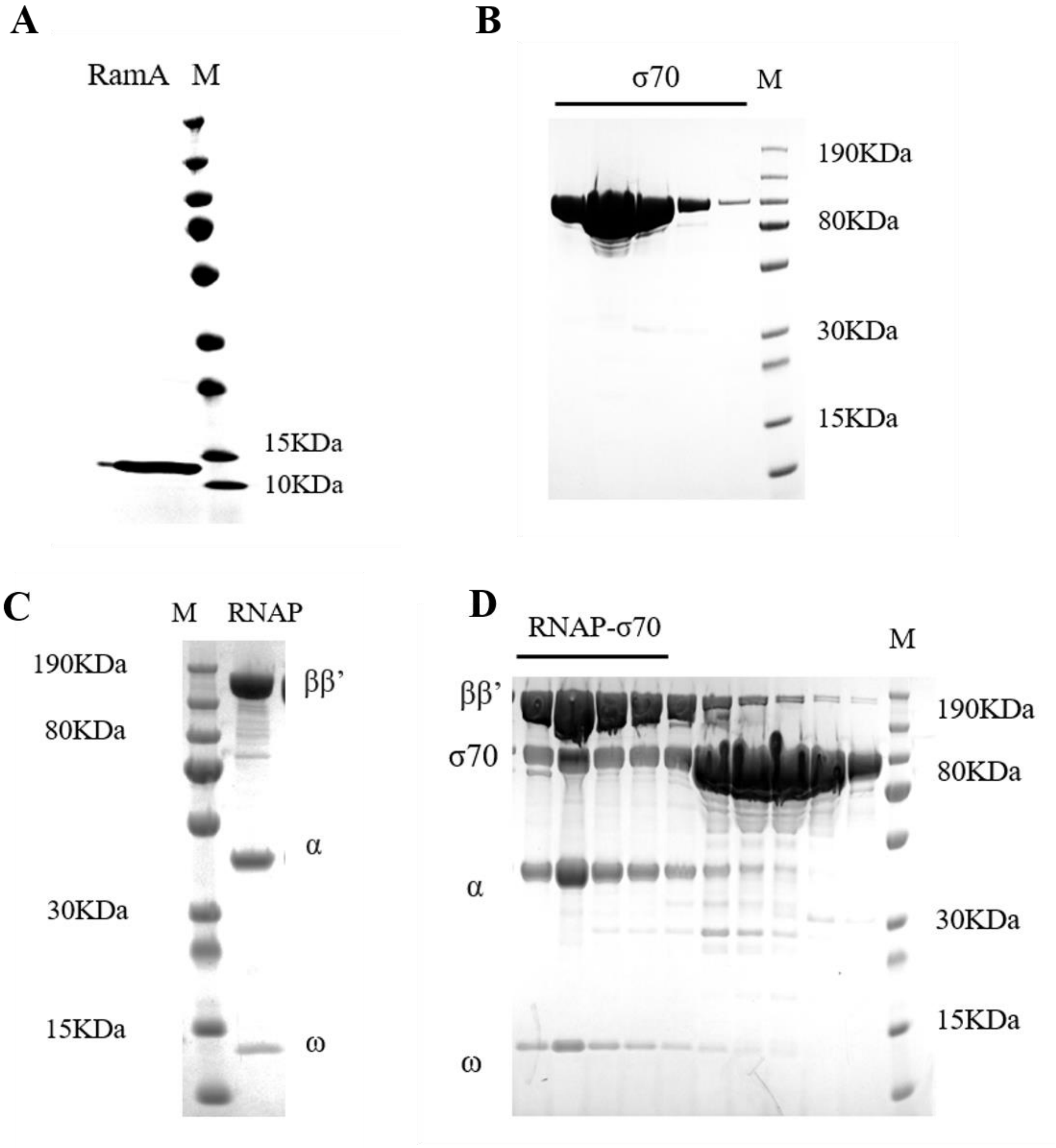

Fig. S11. SDS-PAGE gels of purified proteins: A) RamA, B) σ^{70}, C) RNAP, D) RNAP- σ^{70}

Table S1. Strains and plasmids used in this study

strains vectors or plasmids	genotype or description	Reference or source
E.coli		
MG1655	Escherichia coli K-12 strain	Lab collection
BL21(DE3)	protein expression strain	NEB
K.pnuemonia		
KP22ΔRamA	clnincal isolated multidrug resistance K.pnuemonia, ramA gene knocked out	Lab collection
pOPINF	expression vector Amp^r	Lab collection
pOPINF-RamAWT	pOPONF derivative expressing RamA	This study
pOPINF-RamAM4	pOPONF derivative expressing RamA-Y75R/D76R/D84R	This study
pOPINF-RamAM5	pOPONF derivative expressing RamA-H31D	This study
pOPINF-RamAM6	pOPONF derivative expressing RamA-H31D/Y75R/D76R/D84R	This study
pBad18cm	expression vector, Cm^r	Lab collection
pBad18cm-RamAWT	pBad18cm derivative expressing RamA	This study
pBad18cm-RamAM4	pBad18cm derivative expressing RamA-Y75R/D76R/D84R	This study
pBad18cm-RamAM5	pBad18cm derivative expressing RamA-H31D	This study
pBad18cm-RamAM6	pBad18cm derivative expressing RamA-H31D/Y75R/D76R/D84R	This study
pHSG398	high copy number vector, Cm^r	Takara
pHSG398-RamAWT	pHSG398 derivative expressing RamA	This study
pHSG398-RamAM4	pHSG398 derivative expressing RamA-Y75R/D76R/D84R	This study
pHSG398-RamAM5	pHSG398 derivative expressing RamA-H31D	This study
pHSG398-RamAM6	pHSG398 derivative expressing RamA-H31D/Y75R/D76R/D84R	This study
pGEMABC	encoding full length rpoA, rpoB and rpoC of E. coli	Lab collection
pACYCDuet-omega	encoding full length rpoZ of E. coli	Lab collection
pOPINF-sigma70	encoding full length sigma70 of E. coli	Lab collection
pBBR1MCS-PpacrA-LacZ	Fusion of PacrA promoter and LacZ	This study
pBBR1MCS-PmicF-LacZ	Fusion of PmicF promoter and LacZ	This study
